# Mechanically stable solvent-free lipid bilayers in nano- and micro-tapered apertures for reconstitution of cell-free synthesized hERG channels

**DOI:** 10.1038/s41598-017-17905-x

**Published:** 2017-12-18

**Authors:** Daisuke Tadaki, Daichi Yamaura, Shun Araki, Miyu Yoshida, Kohei Arata, Takeshi Ohori, Ken-ichi Ishibashi, Miki Kato, Teng Ma, Ryusuke Miyata, Yuzuru Tozawa, Hideaki Yamamoto, Michio Niwano, Ayumi Hirano-Iwata

**Affiliations:** 1 Laboratory for Nanoelectronics and Spintronics, Research Institute of Electrical Communication, Tohoku University, 2-1-1 Katahira, Aoba-ku, Sendai, Miyagi 980-8577 Japan; 2 Hang-Ichi Corporation, 1-7-315 Honcho, Naka-ku, Yokohama, Kanagawa 231-0005 Japan; 3 Advanced Institute for Materials Research, Tohoku University, 2-1-1 Katahira, Aoba-ku, Sendai, Miyagi 980-8577 Japan; 4 Department of Chemistry, Graduate School of Science and Engineering, Saitama University, 255 Shimo-Okubo, Sakura-ku, Saitama, Saitama 338-8570 Japan; 5 Frontier Research Institute for Interdisciplinary Sciences, Tohoku University, 6-3 Aramaki-Aza-Aoba, Aoba-ku, Sendai, Miyagi 980-8578 Japan; 6 Kansei Fukushi Research Institute, Tohoku Fukushi University, 6-149-1 Kunimi-ga-oka, Aoba-ku, Sendai, Miyagi 989-3201 Japan

## Abstract

The self-assembled bilayer lipid membrane (BLM) is the basic component of the cell membrane. The reconstitution of ion channel proteins in artificially formed BLMs represents a well-defined system for the functional analysis of ion channels and screening the effects of drugs that act on them. However, because BLMs are unstable, this limits the experimental throughput of BLM reconstitution systems. Here we report on the formation of mechanically stable solvent-free BLMs in microfabricated apertures with defined nano- and micro-tapered edge structures. The role of such nano- and micro-tapered structures on the stability of the BLMs was also investigated. Finally, this BLM system was combined with a cell-free synthesized human *ether-a-go-go-*related gene channel, a cardiac potassium channel whose relation to arrhythmic side effects following drug treatment is well recognized. Such stable BLMs as these, when combined with a cell-free system, represent a potential platform for screening the effects of drugs that act on various ion-channel genotypes.

## Introduction

Cells, the building blocks of life, are encapsulated within cell membranes that regulate ion flow in and out of cells. The cell membrane is composed of a bilayer lipid membrane (BLM), a self-assembled structure of phospholipid molecules, and embedded membrane proteins. Among them, ion channels function as gated pores that permit ions to move across the highly resistive BLMs. Owing to their crucial roles in transmembrane signaling, ion channels have attracted attention not only as primary molecular targets for drug actions but also as major targets for drug-induced side effects^1,2^. Recording ion-channel currents represents an efficient method for investigating the functions of ion channels and screening the drug effects acting on them^3,4^. There are two common approaches for measuring ion channel currents, i.e., patch clamping of natural cell membranes^3^ and the reconstitution of ion channels in the BLM^5,6^. Patch clamping typically involves the use of eukaryotic cells that can express the ion channel of interest on the cell membrane^3,7^. This method requires careful culturing and/or the differentiation of target cells, which can be a lengthy process. For example, 30–90 days are required to produce induced pluripotent stem (iPS) cell-derived cardiomyocytes that are appropriate for drug screening^8^. BLM reconstitution of ion channels is a protein-based method for measuring ion channel currents. The advantage of this system is its versatility in terms of producing target channels, including those prepared by cell-free protein synthesis^9^, in which no cell culturing is needed and pure proteins of various channel genotypes can be produced within a few hours^10–12^. However, the BLM system suffers from low experimental efficiency due to the instability of the BLMs.

The simplest way to enhance the stability of BLMs is to reduce the size of free-standing BLMs by forming them in nanofabricated apertures^13–16^. However, the reduced BLM area of such preparations makes it more difficult to incorporate ion channel proteins, especially those delivered by proteoliposomes^17,18^. To address this issue, several groups have fabricated relatively large apertures (several tens of micrometers in diameter) with tapered-wall edges in attempts to form stable BLMs^18–24^. The tapered-wall appears to be suitable for decreasing perturbation in the topology of BLMs around the point of contact with the aperture edge. Mechanically stable BLMs with integrated protein ion channels have been formed in nano-tapered apertures fabricated in nanometer-thick silicon nitride (Si_3_N_4_) septa^22–24^ and in micro-tapered apertures fabricated in micrometer-thick photosensitive polymer films^18^. However, the issue of why both nano- and micro-tapered edge structures result in the formation of more stable nanostructured BLMs remains unclear, in that the roles of nano- and micro-tapered edges in the formation of stable BLMs are unknown. This represents a difficult problem to solve, since reproducibly fabricating microapertures that contain defined edge structures both at the nano- and micro-meter level is currently a difficult task.

As a nanomaterial that suspends BLMs, silicon nitride (Si_3_N_4_) offers compatibility with well-established fabrication technology and has a high mechanical stability^25^. Aperture formation in nanometer-thick Si_3_N_4_ septa suspended over Si substrates has been intensively studied in attempts to construct a platform for investigating translocation events and single-molecular analysis, such as sequencing DNA bases^25–28^, the translocation of individual proteins^29^ and particles^30^, oligomerization of single proteins^31^ and opening-closing behaviors of single ion-channels embedded in BLMs^17,22–24,32,33^, although reproducibly producing apertures with well-defined edge structures in highly tensile Si_3_N_4_ continues to be a challenge^25,27^. Here we report on a reproducible process for forming a set of apertures with different nano- and micro-edge structures in Si_3_N_4_ septa on which a thin SiO_2_ layer was deposited. Solvent-free BLMs were formed in the apertures with different edge structures and their mechanical and static stability was examined. Combining the BLM system with cell-free protein synthesis was also investigated using the wild-type human *ether-a-go-go-*related gene (hERG) potassium channels as a representative example.

## Results

### Reproducible formation of microapertures with tapered edges

**Figure 1:**
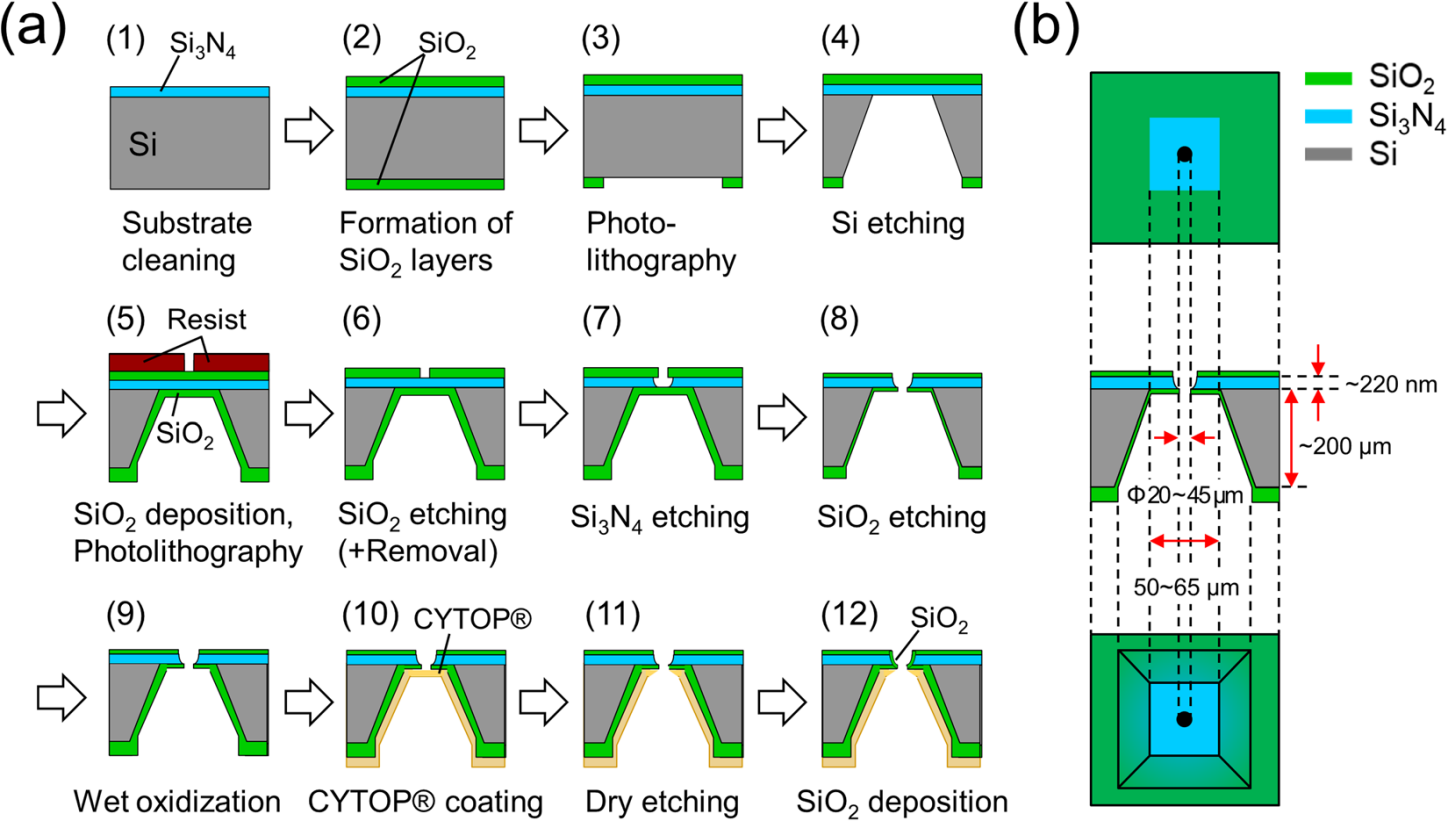
Fabrication of microapertures in Si chips. (**a**) Procedure for fabricating Si chips with microapertures, and (**b**) schematics of fabricated Si chips after Step (8) in (**a**). (top) Top view from the Si_3_N_4_ side, (middle) a cross-sectional view, (bottom) bottom view from the Si side.

**Figure 2:**
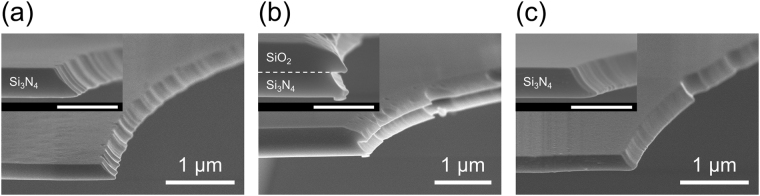
FE-SEM images around the very edge regions of the apertures. Bird’s-eye views and cross-sectional views (each inset; scale bar, 500 nm) are shown. During Step (6) in Fig. 1(a), (**a**) SiO_2_ was sequentially etched in BHF and HF, (**b**,**c**) SiO_2_ was etched in BHF. The chips A and B were analyzed after Step (9). In the case of C, the SiO_2_ layer on the Si_3_N_4_ layer was removed after Step (8) and a thin layer of SiO_2_ was sputtered again after Step (9). The conditions for the final SiO_2_ sputtering were the same as Step (12).

Figure 1 shows the procedure used to fabricate microapertures in Si_3_N_4_ septa suspended over Si, which is modified from the procedure described in ref.^34^. In addition to Step (7) in which apertures were isotropically wet-etched in a Si_3_N_4_ layer, the preceding process (Step (6)) was also found to be critical, not only for producing apertures without cracks but also for determining the edge structures both at the nano- and micro-meter levels. In Step (6), a layer of SiO_2_ on the Si_3_N_4_ layer was wet-etched after photolithographic patterning. When hydrofluoric acid (HF) was used for etching the SiO_2_, the edge of the apertures appeared to be rough and fragile (Fig. S1). The thickness of the Si_3_N_4_ edge was much thinner than that of the original Si_3_N_4_ layer (~220 nm). Consequently, severe cracks were observed around the apertures for most of the fabricated chips and the yield of properly fabricated chips was less than 10% up to only Step (7). Although the apertures fabricated with this procedure were found to be suitable for formation of highly stable BLMs^22,24^, the low fabrication yield would be problematic for future applications, including their use in high-throughput screening. When buffered hydrofluoric acid (BHF) was used as a SiO_2_ etchant, the SiO_2_ layer remained on the Si_3_N_4_ layer and a much thicker septum (~400 nm) was formed (Fig. 2(b)). The yield of the fabrication process up to Step (12) was drastically improved to 87% (n = 36). However, both the Si_3_N_4_ and SiO_2_ layers were wet-etched isotopically, which resulted in the formation of a somewhat complicated double-tapered edge: tapered edge in the Si_3_N_4_ layer (bottom) and that in the SiO_2_ layer (top). An intermediate edge structure (Fig. 2(a)) was obtained when the SiO_2_ layer was etched sequentially in BHF and HF during Step (6). The thickness of the septum was ~230 nm, which was almost the same thickness as that of the Si_3_N_4_ layer, suggesting that only a thin SiO_2_ layer remained on the Si_3_N_4_ layer. The edge of the Si_3_N_4_ layer was smoothly tapered with an angle of 45 degrees. This process was also reproducible and the overall fabrication yield up to Step (12) was 82% (n = 63). Thus, a reproducible fabrication process for forming apertures with defined nano-edge structures was developed.

### Nano- and micro-scale structures around apertures

**Figure 3:**
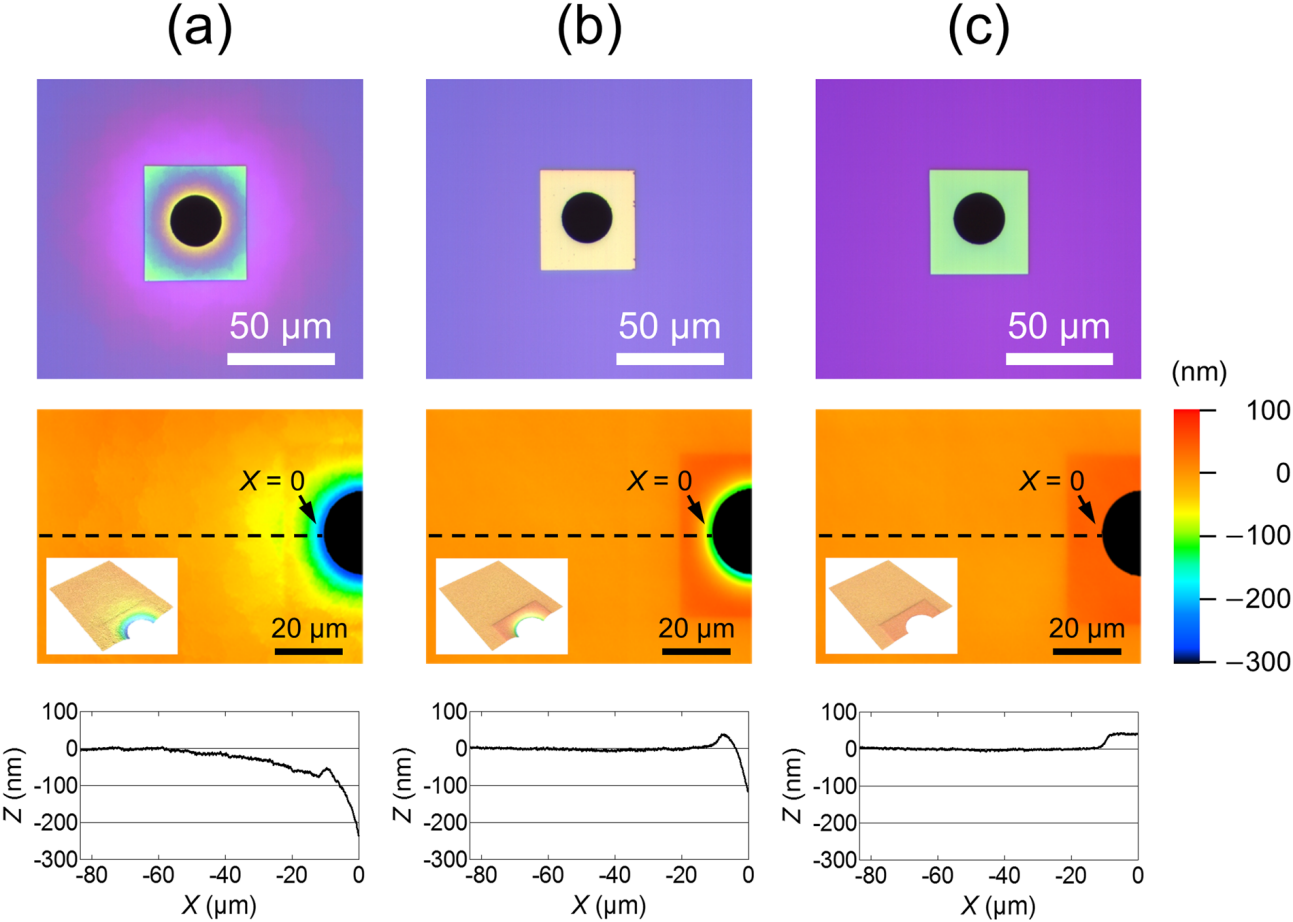
Micrometer-range structure around the aperture edges. (top) Photomicrographs of the apertures from the top. (middle) Laser scanning confocal microscopic images around the edge of the apertures. Bird’s-eye view is shown in the inset. (bottom) Height profiles along the dashed line on the images in the middle. (**a–c**) The micrometer-range structures of the chips A**–**C. Note that all the samples were prepared following the same procedures as described in Fig. 2.

**Figure 4:**
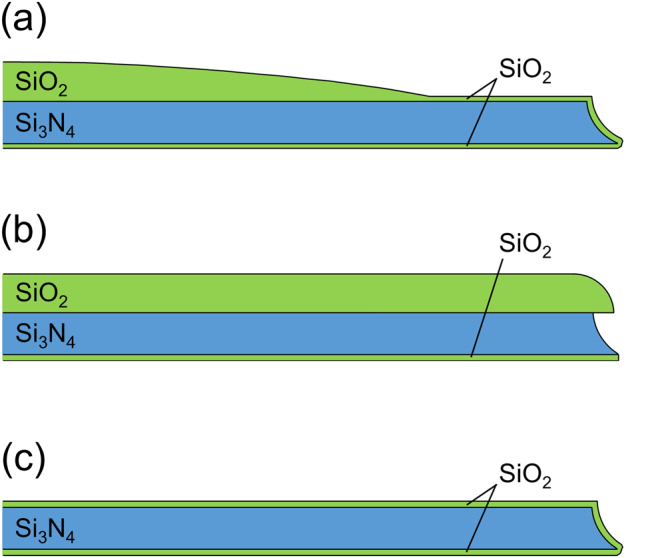
Schematic illustration of tapered-edge structures of Apertures A–C. (**a–c**) Schematic illustration of tapered-edge structures of Apertures A**–**C based on the FE-SEM and laser scanning confocal microscopic images.

We next investigated the structure of the aperture edge in a micrometer scale using 3D laser scanning confocal microscopy. Figure 3 shows top view images, topographic 2D and 3D images, and height profiles of the top surface of the chips around the apertures which were fabricated based on various Step (6) conditions. All chips were analyzed from the Si_3_N_4_ side. When the SiO_2_ layer was sequentially wet-etched in BHF and then HF during Step (6), the top surface of the fabricated chip gradually decreased to ~240 nm within several tens of μm towards the edge of the aperture (Fig. 3(a)). The chip surface tapered off smoothly except for the point around *X* = −10 μm, near the border of a square Si_3_N_4_ window. A top view image of the chip showed a circular interference pattern of SiO_2_ within several tens μm from the aperture, indicating that the thickness of the SiO_2_ layer gradually decreased as it approached the aperture edge. The Si chips thus fabricated had a septum with a long micrometer-scale gradient and a nano-tapered edge (Fig. 4(a)). When the SiO_2_ layer was etched in BHF during Step (6), the top surface of the chip was near flat, except for the region near the aperture edge (Fig. 3(b)). The Si_3_N_4_ layer was single color, indicating that the thickness of the SiO_2_ layer was uniform. The Si chip thus fabricated had a thick and flat septum with a double-tapered nano-edge (Fig. 4(b)). Hereafter, apertures with edge structures shown in Fig. 4(a) and (b) are referred to as “Aperture A” and “Aperture B”, respectively. Since these two apertures had different edge structures on both the nanometer- and micrometer-scales, we also fabricated another edge structure for purposes of comparison. The third structure (Aperture C) was produced by removing the SiO_2_ layer on the Si_3_N_4_ septum after Step (8). This process yielded a Si_3_N_4_ septum with a thickness of ~250 nm and a single-tapered nano-edge (Fig. 2(c)). The top surface of the chip having Aperture C was flat except for a step at the border of the Si_3_N_4_ window (Fig. 3(c)). Although the thick SiO_2_ layer was removed after Step (8), the Si chips for BLM measurements were further treated with Steps (9)–(12), yielding chips whose Si_3_N_4_ layers were covered with a thin layer of SiO_2_ (Fig. S2). As schematically shown in Fig. 4, the nanostructure of Aperture C at the very edge region was similar to that of Aperture A, while a similar flat microstructure was observed with Apertures B and C. Only Aperture A had a micro-tapered structure in addition to a nano-tapered edge. Thus, three sets of microapertures with different nano- and micro-tapered structures were reproducibly fabricated in Si_3_N_4_ septa suspended over Si.

### Evaluation of the mechanical and static stability of BLMs formed in the apertures

Solvent-free BLMs were formed in Apertures A–C and the stability of the BLMs were investigated in terms of both static and mechanical stability. Static stability was evaluated based on membrane lifetime and tolerance to an applied high voltage of ±1 V. Mechanical stability was evaluated based on tolerance to an applied centrifugal force (55 × *g*) and tolerance to mechanical shock during the movement of the solution-air interface over the aperture. Moving the solution-air interface mimics conditions of solution exchange, and was performed by the repeated gentle aspiration of aqueous solutions from both compartments and the reinjection of the same volumes into the compartments^18^. The application of a centrifugal force to BLMs has been reported to be useful for enhancing the probability of channel incorporation, but unstable BLMs are broken by such an applied centrifugal force^34^. Therefore, the mechanical stability of the BLMs was evaluated by examining the tolerance of the membrane to two critical steps in BLM reconstitution systems, i.e., channel incorporation and solution exchange.

**Figure 5:**
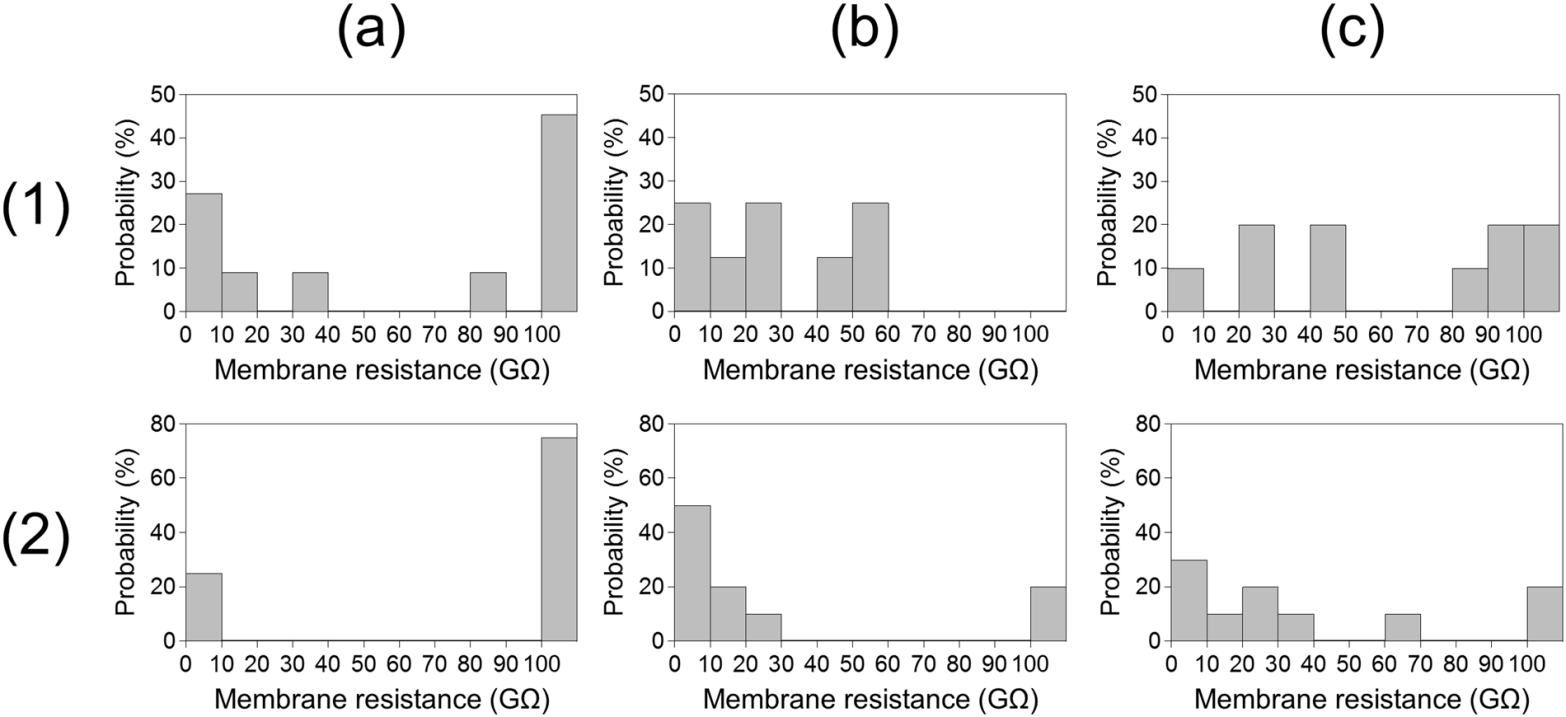
Probability histograms of membrane resistance after BLMs in Apertures A–C were subjected to centrifugation and aspiration cycles. (1) Probability histograms of BLM resistance after being subjected to a centrifugal force (55 × *g* for 10 min) and (2) probability histograms of BLM resistance after being subjected to 20 aspiration cycles. (**a**) BLMs in Aperture A, (**b**) BLMs in Aperture B, and (**c**) BLMs in Aperture C. All the BLMs had a resistance higher than 100 GΩ 10 min after their formation. The probability of BLMs retaining a resistance higher than 100 GΩ in each histogram corresponds to the values shown in Table 1.

Table 1 summarizes the mechanical and static stability of solvent-free BLMs formed in Apertures A–C, together with the probability of BLM formation. BLMs were most reproducibly formed in Aperture A, which had a micro-tapered structure in addition to a nano-tapered edge. These BLMs showed the highest mechanical and static stability, in terms of tolerance to an applied centrifugal force, tolerance to aspiration cycles, tolerance to applied high voltages (±1 V), and average membrane lifetime. Although Aperture C had nearly the same nano-edge structure as Aperture A (Fig. 4), BLMs formed in Aperture C were much less stable than those in Aperture A. The stability of BLMs in Aperture C was similar to that of BLMs in Aperture B, which had a similar flat microstructure as Aperture C. The BLM resistance after the centrifugal force treatment and aspiration cycles tended to be lower in Aperture B than in Aperture C (Fig. 5), reflecting possible differences in the nano-tapered edge structures, i.e., a double-tapered edge for Aperture B and a single-tapered edge for Aperture C. BLMs formed in these two apertures showed average lifetimes of several hours, the longest being 20 days, and tolerance to high voltage (±1 V), which were superior to the corresponding values reported for solvent-free BLMs^33,35^. Thus, the nano-tapered edge of the aperture was found to be useful for forming stable, solvent-free BLMs. However, more stable BLMs were reliably formed in Aperture A, showing the highest tolerance to mechanical stress and an average lifetime more than twice as long as that of BLMs formed in Apertures B and C. It is noteworthy that half of the BLMs in Apertures A survived 40 aspiration cycles (Fig. S3)^18^, exhibiting similar mechanical stability to previously reported BLMs containing *n*-hexadecane, although the use of an organic solvent has been a common approach for improving the stability of BLMs^10–24^.

**Table 1:** Stability of solvent-free BLMs formed in Apertures A–C. The mechanical and static stability of solvent-free BLMs formed in Apertures A–C, together with the probability of BLM formation.

Aperture type	Probability of BLM formation	Probability of tolerance^**a**^			Lifetime^**e**^	
		centrifugal force^**b**^	aspiration cycles^**c**^	applied voltage^**d**^	average	maximum^**f**^
A	85% (n = 48)	45% (n = 11)	75% (n = 12)	100% (n = 13)	9.8 ± 3.1 h (n = 13)	46 h
B	58% (n = 36)	0% (n = 8)	20% (n = 10)	100% (n = 5)	4.9 ± 2.3 h (n = 5)	20 days
C	59% (n = 44)	20% (n = 10)	20% (n = 10)	100% (n = 4)	3.5 ± 1.8 h (n = 4)	28 h

^**a**^Probability of BLMs maintaining a membrane resistance higher than 100 GΩ after centrifuging, aspiration cycles, and high voltages. Only the BLMs whose resistance at 10 min after their formation was higher than 100 GΩ were evaluated. The diameter of the apertures was in the range from 20 to 30 μm. ^**b**^Conditions for centrifugation: 55 × *g* for 10 minutes. ^**c**^Number of aspiration cycles: twenty. ^**d**^A square voltage waveform was applied as follows. Applied potential was first switched from 0 to +1 V and held at +1 V for 0.5–1 min, and the potential was then switched to −1 V and held at −1 V for 0.5–1 min. Finally, the potential was set back to 0 V. ^**e**^Lifetime was defined as the duration for which the membrane resistance was higher than 100 GΩ. ^**f**^These maximum values were not included in the calculation of the average.

### Single-channel recordings of cell-free expressed hERG channels

**Figure 6:**
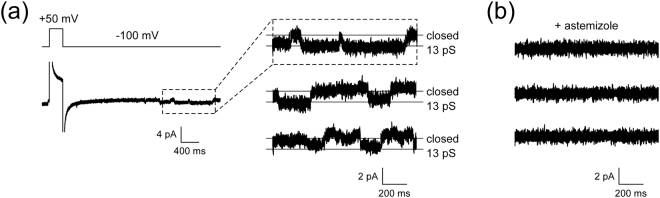
Examples of single-channel currents of cell-free synthesized hERG channels before and after the addition of astemizole. (**a**) Typical single-channel currents recorded at –100 mV after a prepulse of +50 mV. An expanded current trace is shown in the top trace on the right. Three representative currents obtained in the same manner from the same BLM are shown. (**b**) Current traces after the addition of astemizole, which was added to the *trans* compartment. The final concentration of astemizole in the *trans* compartment was 1 μM. The diameter of the aperture was 45 μm.

Finally, we combined the present BLM system with cell-free protein synthesis. A cell-free synthesized hERG channel was prepared (Fig. S4) and incorporated into the BLMs formed in Aperture A and the channel activity of the preparation was investigated. The hERG channel is a voltage-gated potassium channel that is essential for normal electrical activity in the human heart^36,37^. This channel has attracted considerable interest, because a diverse group of drugs have been found to adversely block hERG channels, which sometimes induces life-threatening arrhythmias^36,37^. It has recently been proposed that such drug-induced arrhythmia may be related to disease-causing genetic variants in *KCNH2*, the gene that encodes the hERG channel^38^. The BLMs formed in Aperture A were centrifuged repeatedly with proteoliposomes containing the cell-free synthesized wild-type hERG channel until hERG channel currents were observed or the BLMs were broken. Channel activities of the hERG channels, including single and multiple channels, were recorded from 31% of BLMs (19 out of 61 BLMs) usually 10–15 min after the centrifugation. As shown in Fig. 6, clear stepwise currents corresponding to single-channel activities were recorded. A total of 65 single-channel events were observed from 12 BLMs. The average single-channel chord conductance was 13.0 ± 0.2 pS (mean ± SEM) from the 12 BLMs. This conductance level was very close to those (11–13 pS in 120 mM KCl) reported for hERG channels expressed in *Xenopus* oocytes^39^ and BLMs containing hERG channels isolated from cell expression systems^23,24,40^. We then added an antihistamine astemizole, which has been withdrawn from the market due to its adverse inhibitory effect on hERG channels^36^. The addition of astemizole completely blocked the channel activities, demonstrating that the cell-free synthesized hERG channel is also blocked by this compound. Thus, the cell-free synthesized hERG channels incorporated into the present BLM system exhibited similar channel properties to the hERG channels produced by cell expression systems, in terms of single-channel conductance and sensitivity to astemizole. However, the channel activities of the hERG channels usually persisted for only 30–60 min, which made it difficult to examine the stability of the BLMs that contained hERG channels. The average number of 10-min centrifugations at 900 rpm required for observing the hERG channel activities was 1.6 ± 0.2 (n = 19), which showed no significant difference from the average number (2.1 ± 0.2, n = 35) of centrifugations that resulted in BLM rupture. In the absence of proteoliposomes containing hERG channels, a significantly larger number (4.1 ± 0.7, n = 7) of the centrifugations was required before the BLMs were broken. These results suggest that proteoliposome fusion can cause fatal damage to the BLMs, leading to a high risk of BLM rupture (57%, 35 out of 61 BLMs) even for the highly stable BLMs in Aperture A.

## Discussion

We established a reproducible process for fabrication of microapertures with defined nano- and micro-edge structures in nanometer-thick Si_3_N_4_/SiO_2_ septa. Through the comparison of static and mechanical stability of BLMs formed in the apertures, it was found that both nano-tapered and micro-tapered edge structures were important in forming stable solvent-free BLMs, however, a micro-tapered edge structure was more crucial for the mechanical stability of a BLM. We previously reported on the formation of mechanically stable solvent-free BLMs in apertures that were fabricated in Si chips using HF as the etchant for Step (6) (Fig. 1(a))^22,34^. The edge of the apertures thus formed had similar micro-tapered structures in addition to nano-tapered edges (Fig. S5), which also supports the present observations. Considering the fact that stable BLMs in micro-tapered apertures were formed in micrometer-thick photosensitive resin only in the presence of organic solvent^18–21^, the nano-tapered edge structure would be essential for the formation of solvent-free BLMs. The additional micro-tapered structure would be expected to improve tolerance to mechanical stimuli which might distort the shape of the BLMs on a micrometer-scale. For example, it has been reported that BLMs easily form bulges upon application of stimuli, such as osmotic pressure, an applied force and hydrostatic pressure^41–43^. If the present BLMs also bulged to a similar extent as phosphocholine BLMs in an aperture (ϕ~1 mm)^44^, the bulging depth was estimated to be ~3.5 μm for the BLMs in the present aperture (ϕ~25 μm). We speculate that the gradual long-tapered edge is more suitable to smoothly support BLMs that are bulging under conditions of mechanical stimuli such as an applied centrifugal force and the movement of water. Another possibility is based on a theoretical study reported by White^5,45^, in which he modeled the shape of an annulus of a lipid solution surrounding BLMs that were in contact with aperture walls. For the formation of stable BLMs, based on his modelling, the aperture-annulus contact angle should be small or lipids should drift along the aperture wall appropriately, which can be achieved by the use of a very thin septum or a countersunk aperture, respectively. When we extended his findings to the present system of solvent-free BLMs, Aperture A has a very thin septum with a countersunk structure with head and body diameters of ~150 and ~25 μm, respectively, while Aperture C has only a very thin septum. Therefore, appropriate lipid drift would be expected for Aperture A, leading to the higher mechanical stability of BLMs in Aperture A.

By using the mechanically stable solvent-free BLMs formed in Aperture A, we also succeeded in reconstitution and single-channel recording of a cell-free expressed wild-type hERG channel. The channel activity was inhibited by astemizole, whose adverse effect on the hERG channel is well recognized, demonstrating the potential of the present system as a platform for assessing risks of drug side effects acting on hERG genotypes. The advantage of the present BLMs combined with cell-free expression system is its broad applicability to various ion channel genotypes including disease-causing genetic variants. Considering that a relationship has been implied between the hERG channel genotypes and drug-induced arrhythmia^38^, extending the present approach to various hERG genotypes has the potential to serve as a new screening platform for assessing the potential risks of drug side effects acting on hERG channels of patients.

## Methods

### Materials

Chloroform solutions of L-α-phosphatidylcholine (PC, from egg), L-α-phosphatidylethanolamine (PE, transphosphatidylated (egg)), were purchased from Avanti Polar Lipids (Alabaster, AL). Cholesterol (Chol) was obtained from Wako Pure Chemicals (Osaka, Japan) and recrystallized three times from methanol prior to use. Astemizole was obtained from Sigma-Aldrich (St. Louis, MO). CYTOP® (CTL-809M) and buffered hydrofluoric acid (BHF) (LAL 400 SA) were purchased from Asahi Glass Co., Ltd. (Tokyo, Japan) and Stella Chemifa Corporation (Osaka, Japan), respectively. (Tridecafluoro-1,1,2,2-tetrahydrooctyl)dimethylchlorosilane (PFDS) was obtained from Gelest (Morrisville, PA). Anhydrous toluene was purchased from Wako Pure Chemicals.

### Fabrication of apertures in Si chips

Microapertures A–C were fabricated in Si chips, according to the procedure described in ref.^34^ with several modifications. A cleaned FZ Si (100) wafer (≥9000 Ω cm, 200 μm in thickness), one side of which was coated with an ~220 nm thick Si_3_N_4_ layer (Semitec, Chiba, Japan), was thermally oxidized in a stream of dry oxygen, and the Si_3_N_4_ side was then coated with SiO_2_ by RF sputtering at ~300 °C. The Si side was photolithographically patterned, and anisotropically etched in a tetramethylammonium hydroxide (TMAH) solution at 90 °C. SiO_2_ was then sputtered onto the Si side to cover the bare Si_3_N_4_ and Si surfaces, which were exposed after the anisotropic etching. After the photolithographic patterning of circles from the Si_3_N_4_ side, the SiO_2_ layer sputtered on the Si_3_N_4_ was etched in BHF and then in 5% hydrofluoric acid (HF). In some cases, the SiO_2_ layer was etched in only BHF. The photoresist was then removed, and the exposed Si_3_N_4_ layer was isotropically etched in 85% phosphoric acid at 150 °C. The SiO_2_ layer beneath the etched holes was removed by treatment with a 5% HF solution to form microapertures. Next, a SiO_2_ layer was formed on the surface of the Si chips using wet thermal oxidation for 1 h. A layer of a SU-8 3010 photoresist (MicroChem Corp., Westborough, MA) was spin-coated onto a dummy wafer, and the Si chip was placed on the SU-8 3010 layer from the Si_3_N_4_ side. Fluoropolymer CYTOP® was drop-casted to cover the whole Si chip. The dummy wafer with the Si chip was turned upside down. The wafer was then spun at 1,000 rpm for 20 s, and air-dried for 30 min. After baking at 90 °C for 10 minutes, the chip was removed from the dummy wafer in SU-8 Developer (MicroChem Corp.) at 100 °C and baked at 200 °C for 1 h. The CYTOP® layer clogged in the microapertures was removed by exposing the chip to oxygen plasma (1 h, 500 W; Plasma Cleaner V-1000, Yamato Scientific Co., Ltd., Tokyo, Japan) from the Si_3_N_4_ side. Finally, a thin layer of SiO_2_ was sputtered on the chip from the Si_3_N_4_ side at ~300 °C.

### Characterization of Si chips

Nano- and micro-scale profiles around the apertures after the wet thermal oxidation were examined. The Si_3_N_4_ side of the Si chip was first coated with a thin layer of Pt using an ion coater (MSP-10, Vacuum Device Co, Ltd., Ibaraki, Japan). A 3D profile of the Pt-coated surface was obtained with a 3D laser scanning confocal microscope (VK-X250/260, KEYENCE Corporation, Osaka, Japan). The nanostructure around the very edge of the apertures were observed using a SU-8000 field emission scanning electron microscope (FE-SEM) (Hitachi High-Technologies Corporation, Tokyo, Japan).

### Preparation of proteoliposomes containing hERG channel protein

The recombinant hERG channel protein (UniProt/SWISS-PROT accession no. Q12809) that is encoded in the human genome was produced using a wheat germ cell-free translation system in the presence of lipid vesicles (liposomes). PC-PE-Chol-liposomes, consisting of 70% PC (w/w), 10% PE, and 20% Chol, were freshly prepared immediately prior to use, as described previously^46^. Plasmid, pYT08-co_hERG, was constructed as the template for *in vitro* mRNA synthesis. For the pYT08-co_hERG, a synthetic DNA encoding a hERG ORF in which the codon usage is optimized to that of the wheat germ translation system was custom synthesized by Eurofins Genomics (Tokyo, Japan) and cloned into SpeI and SalI sites of the pYT08 vector^46^. The nucleotide sequence of the optimized hERG channel has been deposited in GenBank under accession no. LC279614. The mRNAs synthesized from the constructed plasmid were then used in the cell-free translation system in the presence of liposomes^46^. The reaction mixtures were then centrifuged at 20,000 × g for 20 min at 4 °C, and the supernatant and the precipitate were separately collected. The precipitate containing the hERG channel-liposome complex was suspended in Buffer A, containing 30 mM HEPES-KOH (pH 7.8) and 100 mM potassium acetate, in the same volume of translation reaction mixture, and the suspension was centrifuged at 20,000 × g for 20 min at 4 °C. The precipitate was separately collected. This procedure was repeated twice, and the final precipitate was resuspended in buffer A and stored at –80 °C until used. To verify the production of the hERG channel and its purity, 1/100 volume from each fraction were analyzed by SDS-PAGE, followed by visualization by Coomassie brilliant blue staining.

### BLM formation, evaluation of mechanical stability of BLMs, and protein incorporation

The fabricated Si chip was silanized by treatment with 2% (v/v) PFDS in anhydrous toluene for 6 h at room temperature in a nitrogen-filled glove box. Solvent-free BLMs were formed across Apertures A–C by folding up two lipid monolayers without a coating of *n*-hexadecane around the apertures, as described in ref.^34^. A 10–30 μL portion of a lipid solution (2 mg/mL PC:PE:Chol = 7:1:2 (w/w) in chloroform/*n*-hexane (1:1, v/v)) was used for BLM formation. Although BLMs formed by the folding method frequently require a precoating with hydrocarbons (*n*-hexadecane, squalene, etc.) around the apertures^35^, Apertures A–C, having nano-tapered edge structures, led to formation of BLMs in the absence of hydrocarbons. The BLMs were formed in symmetric recording solutions containing 120 mM KCl and 10 mM HEPES (pH 7.2 with KOH). The probability of BLM formation was defined as the percentage of BLMs whose resistance measured 10 min after their formation was higher than 100 GΩ. The resistance of the BLMs was calculated based on the currents observed at +100 mV and −100 mV.

To determine the tolerance of the preparation to aspirations cycles, ~1 mL portions of *cis* and *trans* solutions were gently aspirated and reinjected, and membrane resistance was measured every 10 aspiration cycles. The aspiration cycle experiment was carried out by a well-trained operator according to the following rules. Each aspiration and reinjection was performed in 3 and 1 s, respectively, and this cycle was repeated 10 times without intervals. After waiting for 2 min, membrane resistance was then measured. Since the resistance measurement required 80 s, 4 min were required to operate 10 aspiration cycles. These procedures were repeated until the resistance fell below 100 GΩ. The tolerance of the BLMs to centrifugation was investigated using the following procedures. First, the water level of *cis* and *trans* compartments in a Teflon® chamber was adjusted to the top surface of the chamber by adding the recording solutions (a total of 1,700 μL for both compartments). The Teflon® chamber was sealed with a lid and then spun at 1,000 rpm (55 × *g*) for 10 min using a centrifuge (Model 3740, Kubota Corporation, Tokyo, Japan). After waiting for 10 min, BLM resistance was measured. The centrifugal incorporation of hERG channel was performed in a similar manner after the addition of proteoliposomes containing the hERG channels (typically 30 μL) to the *cis* solution, except that the chamber was repeatedly spun at 900 rpm (44 × *g*) for 10 min. When no channel activities were observed, the chamber was again centrifuged at 900 rpm for 10 min. The centrifugation was repeated up to 5 times until hERG channel currents were observed or the BLMs were broken. The *cis* solution containing the hERG proteoliposomes was reused several times as follows: after BLMs were formed in the symmetric recording solutions, the *cis* solution was replaced with the previously used solution containing the hERG proteoliposomes and spun at 900 rpm.

### Current recordings

Current recordings were performed using an Axopatch 200B patch-clamp amplifier (Molecular Devices, Sunnyvale, CA). Signals were on-line filtered at 1 kHz with a low-pass Bessel filter, digitized at 10 kHz, and stored on-line using a data acquisition system (Digidata 1440 and pCLAMP 10.3, Molecular Devices). The currents were off-line filtered at a cut-off frequency of 0.7 kHz, according to ref.^43^. Applied potentials were defined with respect to the *trans* side held at ground.

## Electronic supplementary material

Supplementary Information
