# Supplementary material for: Mechanically stable solvent-free lipid bilayers in nano- and micro-tapered apertures for reconstitution of cell-free synthesized hERG channels
Source: Sci Rep. 2017 Dec 18;7:17736. doi: 10.1038/s41598-017-17905-x (PMC5735097; doi:10.1038/s41598-017-17905-x)
Supplement: Supplementary file 1 — Supplementary Information [file 41598_2017_17905_MOESM1_ESM.pdf]

Supplementary Information

Mechanically stable solvent-free lipid bilayers in nano- and micro-tapered apertures for reconstitution of cell-free synthesized hERG channels

Daisuke Tadaki[1], Daichi Yamaura[1], Shun Araki[1], Miyu Yoshida[1], Kohei Arata[1], Takeshi Ohori[1], Ken-ichi Ishibashi[2], Miki Kato[1], Teng Ma[3], Ryusuke Miyata[1], Yuzuru Tozawa[4], Hideaki Yamamoto[5], Michio Niwano[6] & Ayumi Hirano-Iwata[1,3*]

[1]Laboratory for Nanoelectronics and Spintronics, Research Institute of Electrical Communication, Tohoku University, 2-1-1 Katahira, Aoba-ku, Sendai, Miyagi, 980-8577, Japan. [2]Hang-Ichi Corporation, 1-7-315 Honcho, Naka-ku, Yokohama, Kanagawa, 231-0005, Japan. [3]Advanced Institute for Materials Research, Tohoku University, 2-1-1 Katahira, Aoba-ku, Sendai, Miyagi, 980-8577, Japan. [4]Department of Chemistry, Graduate School of Science and Engineering, Saitama University, 255 Shimo-Okubo, Sakura-ku, Saitama, Saitama, 338-8570, Japan. [5]Frontier Research Institute for Interdisciplinary Sciences, Tohoku University, 6-3 Aramaki-Aza-Aoba, Aoba-ku, Sendai, Miyagi, 980-8578, Japan. [6]Kansei Fukushi Research Institute, Tohoku Fukushi University, 6-149-1 Kunimi-ga-oka, Aoba-ku, Sendai, Miyagi, 989-3201, Japan. Correspondence and requests for materials should be addressed to A.H-I. (email: ayumi.hirano.a5@tohoku.ac.jp)

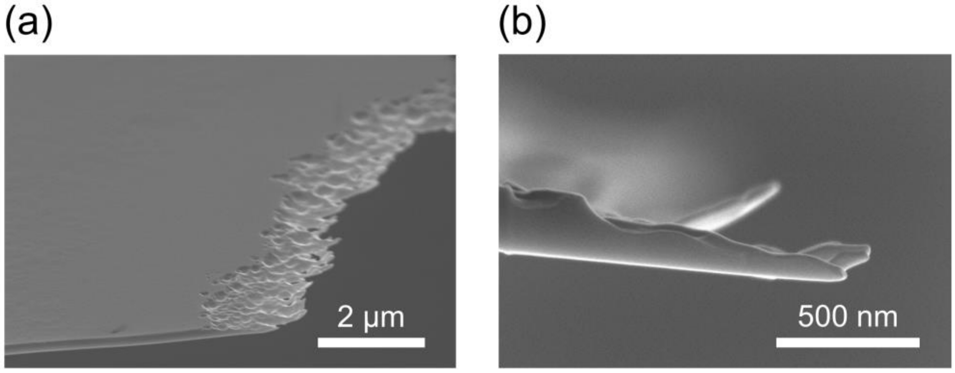

Figure S1. FE-SEM images around the very edge of the apertures. (a) bird's-eye view, and (b) cross-sectional view. The aperture was fabricated based on the process shown in Fig. 1(a) using hydrofluoric acid (HF) as the SiO_2 etchant in Step (6). The structure of the aperture after Step (9) was analyzed.

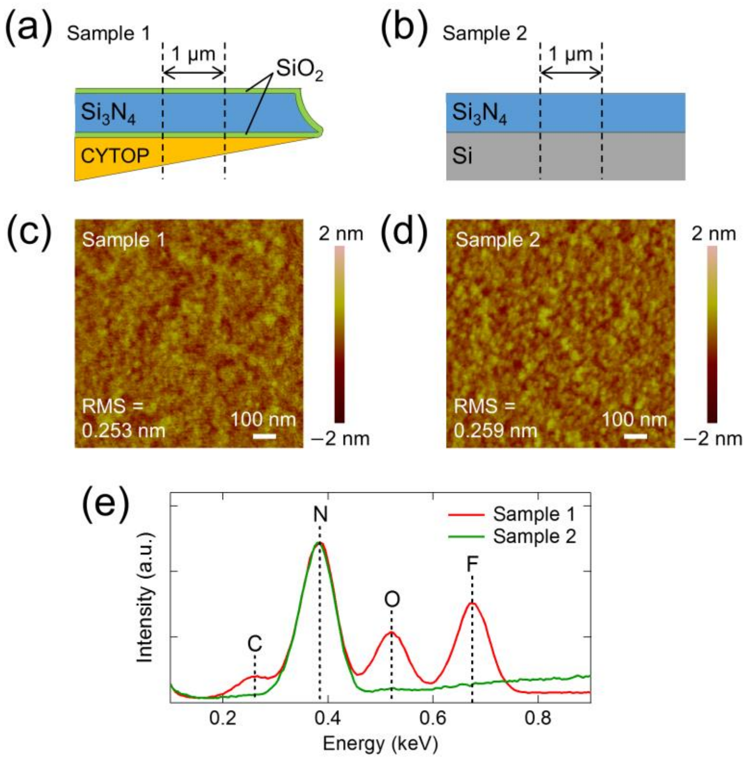

Figure S2. (a,b) Schematic diagrams of Samples 1 and 2. Sample 1 is an edge region around Aperture C. Sample 2 is a Si_3N_4/Si substrate that was used in the fabrication of Apertures A–C. (c,d) AFM images of surface regions in Samples 1 and 2 inside dashed lines depicted in A and B. The surface roughness was observed to be similar for both samples. (e) EDX spectra obtained by measuring the region inside the dashed lines of each sample. Note that the intensity of the nitrogen peak in the green-colored spectrum (Sample 2) was normalized to that in the red-colored spectrum (Sample 1) for comparison. Sample 1 was analyzed after Step (12) in Fig. 1(a).

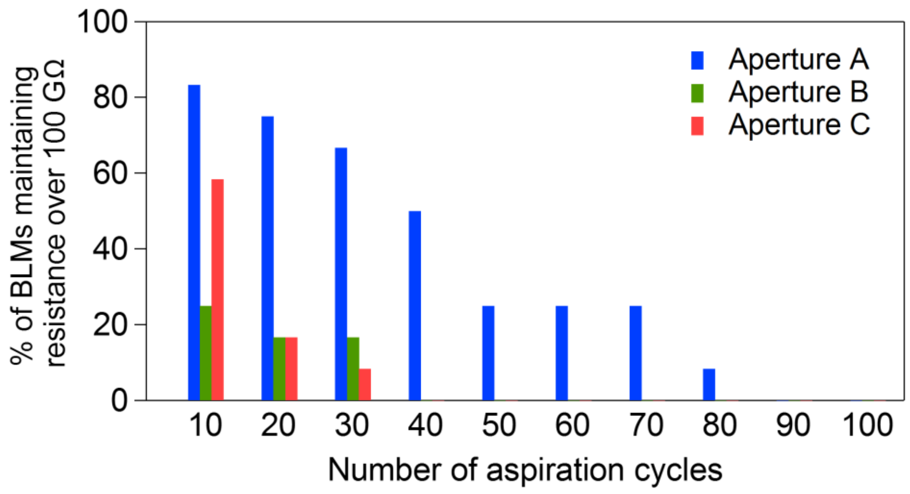

Figure S3. Mechanical stability of BLMs formed in Apertures A–C, quantified as the number of aspiration cycles that can be withstood by the BLMs.

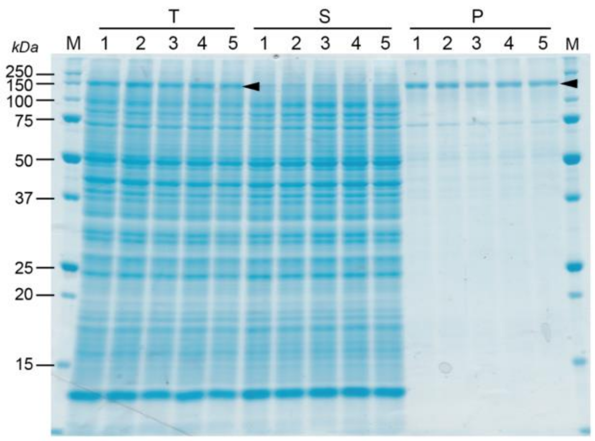

Figure S4. Synthesis of the hERG channel with a cell free system. The hERG channel was synthesized with a wheat germ cell free system in the presence of liposomes, and the translation mixture was then centrifuged at 20,000 × g for 20 min at 4 °C. The total (T), soluble (S), and pellet (P) were subjected to SDS-PAGE and stained with Coomassie brilliant blue. Arrowhead indicates the hERG channel. Molecular weight marker is shown in lane M. Lanes 1 to 5 correspond to five independent translation mixtures.

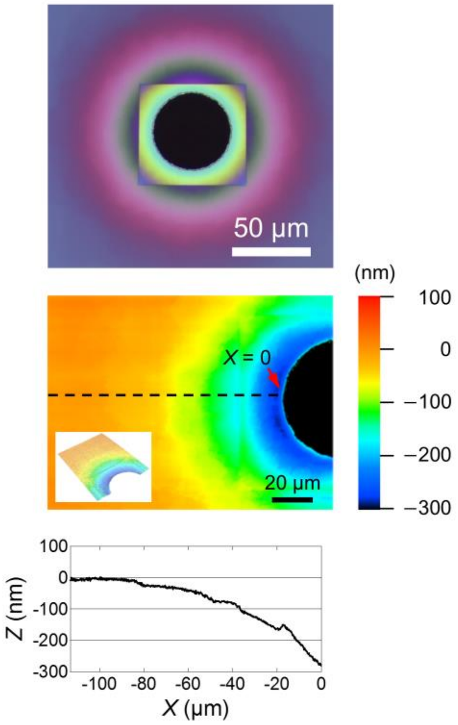

Figure S5. Structure of the aperture edge in a micrometer range. The aperture was fabricated based on the process shown in Fig. 1(a), where HF was used as the SiO_2 etchant during Step (6). The structure of the aperture after Step (9) was analyzed. (top) Photomicrograph of the aperture from the top. (middle) Laser scanning confocal microscopic image around the edge of the aperture. Bird's-eye view is shown in the inset. (bottom) Height profile along the dashed line on the image in the middle.
